# Validation of a Machine-Learning Clinical Decision Aid for the Differential Diagnosis of Transient Loss of Consciousness

**DOI:** 10.1212/CPJ.0000000000200448

**Published:** 2025-02-25

**Authors:** Alistair Wardrope, Melloney Ferrar, Steve Goodacre, Daniel Habershon, Timothy J. Heaton, Stephen J. Howell, Markus Reuber

**Affiliations:** 1 Department of Neurology, Sheffield Teaching Hospitals NHS Foundation Trust, Royal Hallamshire Hospital, Sheffield, United Kingdom;; 2 Division of Neuroscience, Royal Hallamshire Hospital, University of Sheffield, Sheffield, United Kingdom;; 3 Syncope and Postural Tachycardia Syndrome Service, Sheffield Teaching Hospitals NHS Foundation Trust, Royal Hallamshire Hospital, Sheffield, United Kingdom;; 4 Directorate of Acute and Emergency Medicine, Sheffield Teaching Hospitals NHS Foundation Trust, Northern General Hospital, Sheffield, United Kingdom;; 5 Division of Population Health, University of Sheffield, Sheffield, United Kingdom;; 6 Specialised Cancer Services, Sheffield Teaching Hospitals NHS Foundation Trust, Weston Park Cancer Centre, Sheffield, United Kingdom; and; 7 Department of Statistics, School of Mathematics, University of Leeds, United Kingdom.

## Abstract

**Background and Objectives:**

The aim of this study was to develop and validate a machine-learning classifier based on patient and witness questionnaires to support differential diagnosis of transient loss of consciousness (TLOC) at first presentation.

**Methods:**

We prospectively recruited patients newly presenting with TLOC to an emergency department, an acute medical unit, and a first seizure or syncope clinic. We invited participants to complete an online questionnaire, either at home or at time of initial assessment. Two expert raters determined the cause of participants' TLOC after 6-month follow-up. We used independent development and validation samples to train a random forest classifier to predict diagnosis from participants' questionnaire responses and validate classifier performance. We compared classifier performance against penalized linear regression and referrer diagnosis.

**Results:**

We included 178 participants in the final analysis, of whom 46 identified a witness able to complete an additional witness questionnaire. Given low witness recruitment, we developed a classifier based on patient answers only. A classifier trained on 9 items correctly identified 63 of 78 diagnoses (80.8%) (95% CI 70.0–88.5), an increase over the accuracy of initial assessing clinicians who were only able to diagnose 70.5% correctly. Within this, 96% (87.0%–99.4%) of those expertly rated as having syncope were correctly classified by the classifier (classifier sensitivity); 40% (20%–63.6%) of those expertly rated after follow-up as having either epilepsy or functional/dissociative seizures were similarly classified as being nonsyncope (classifier specificity).

**Discussion:**

A machine-learning classifier for differential diagnosis of TLOC has comparable performance in differentiating between 3 main causes of primary TLOC as the current standard of care but is insufficiently accurate in its current form to warrant incorporation into routine care. A system including information from witnesses might improve classification performance.

## Introduction

### Background and Importance

Transient loss of consciousness (TLOC)—spontaneous disruption of consciousness not due to head trauma, with complete recovery^1^—is one of the commonest neurologic concerns in primary/emergency care.^2^ Over 90% is due to syncope, epilepsy, or functional/dissociative seizures ([FDSs]; “psychogenic nonepileptic seizures”).^3,4^ Rapid, accurate diagnosis is vital for appropriate further management. However, 20%–30% of patients are misdiagnosed or mismanaged.^5,6^ Patients who could be reassured that they experienced uncomplicated vasovagal syncope are told they cannot work or drive until expert assessment. Patients who should be investigated by cardiologists are referred to neurologists and vice versa. Investigations to identify life-threatening pathologies are delayed.^2,3,5,7-9^ Misdiagnosis is particularly common in patients with FDSs. The mean interval from first presentation to diagnosis of FDSs is 4 to 7 years,^3^ causing prolonged disability and risking potentially fatal mistreatment.^10,11^

Diagnosis is complicated by the lack of unique distinguishing single clinical features^2,3^ and because interictal investigations are noncontributory in most cases.^9,12^ The optimal extraction of historical information from the patient and any witnesses remains the cornerstone of diagnosis. Previous research suggests that clusters of features can distinguish between causes of TLOC better than individual features.^3,13-15^

At present, taking and interpreting the history require time and specialist expertise, which may not be available in emergency or primary care settings. However, studies based on clinical data such as peri-ictal symptoms suggest that systematic questionnaires may support diagnosis.^3,16^

In previous research, we have shown the diagnostic potential of systematic symptom-reporting questionnaires (captured in the Paroxysmal Event Profile [PEP]) and of witness reporting (the Paroxysmal Event Observer [PEO]) to support differential diagnosis of TLOC.^3,17^ We used machine learning to reduce these extensive questionnaires to the much shorter initial PEP (iPEP). This 36-item iPEP was used to train a machine learning–based diagnostic classifier (the “iPEP classifier”) to discriminate between diagnoses in a cohort of patients with established gold standard diagnoses. In a separate validation sample, this classifier accurately diagnosed 74 of 86 patients (86.0%) correctly (100% syncope, 85.7% epilepsy, 75.0% FDSs).^18^

However, such a tool would be of maximal clinical utility at the point of first presentation, and the validity of using a training sample of patients with long-standing, established diagnoses to support diagnoses in a target population of patients newly presenting with TLOC is uncertain.

### Goal of This Investigation

The objective of this study was to develop and validate a patient-completed questionnaire-based machine-learning classifier within the target population (patients first presenting with TLOC), with questionnaires incorporating new items of potential diagnostic utility identified since development of the PEP/PEO.^13^

## Methods

### Setting

We performed this study in a single large teaching hospital in the United Kingdom, with a large adult emergency department (ED) and tertiary neurology and cardiology services.

### Recruitment and Participants

Prospective recruitment took place from February 10, 2022, to January 9, 2023. One team member (D.H.) screened all admissions to the ED and acute medical unit (AMU) for presentations with TLOC and all new referrals to neurology and cardiology departments for TLOC, according to the criteria stated further.

#### Inclusion Criteria

1. Patients first presenting with TLOC.
2. Referred to secondary care for diagnostic evaluation OR given firm diagnosis of syncope in accordance with European Society of Cardiology (ESC) guidelines for syncope presentations not requiring further investigation.
3. Adults older than 16 years.
4. Able to complete questionnaires independently.
5. Sufficient English language ability to complete questionnaires without support.

#### Exclusion Criteria

1. Unable to give informed consent to participation in research.
2. Unable to complete questionnaires independently.
3. Previous specialist (neurologic or cardiologic) assessment of TLOC.
4. Secondary cause of TLOC identified.

We invited participants either in person (during their ED or AMU attendance) or before specialist assessment, sending participant information sheets about the study to all individuals identified as eligible.

We asked participants to identify a witness to their TLOC and to share with them a separate information sheet regarding the study. We sought independent consent from witnesses to participate.

### Sample Size

#### Development

There are no simple rules for calculating sample sizes for machine learning with random forest (RF) classifiers. Although the RF approach is optimized for classification problems in low-n high-p settings,^19^ performance improves with increased sample size.^20,21^ We previously demonstrated robust performance of an RF classifier for this problem in a training set of 163 participants.^18^ Simulating classification performance using our previous study data demonstrated that classifier accuracy increased progressively with a sigmoidal distribution flattening out at 40–50 participants. To ensure that we captured enough participants with a sufficiently certain diagnosis to allow inclusion in the analysis, we aimed to recruit 100 participants for the training stage of this study.

#### Validation

We consider the primary clinical problem to be one of separating “cardiologic” (syncope) presentations from “neurologic” (epilepsy or FDSs). In our pilot study, the iPEP classifier had a sensitivity for syncope of 100% (95% CI 86.7%–100%). Demonstrating sensitivity for syncope within the previously determined 95% CI (>86.7%) requires (for 1-tailed α = 0.05 and β = 0.9) 42 participants with syncope in the validation sample.^22^ Previous studies suggest a prevalence of syncope in our target population of approximately 50%.^23^ This gives a total validation sample size of 84. A study of ED attendances with suspected seizures in our target population found that 14.3% could not be given a firm diagnosis of a primary TLOC cause (1.1% unknown diagnosis, 9.9% acute symptomatic seizure, 3.3% missing data).^24^ We, therefore, adjusted our target to 98 participants to ensure recruitment-sufficient numbers with clear diagnoses. Adjusting for loss to follow-up (estimated 6%^25^) provides a final validation sample size of 105 participants.

### Study Instruments

#### PESQ and PEWQ

We used 2 brief questionnaires derived from previous development work^18^ and subsequently published reviews^13,26^: one for patients themselves and one for witnesses if available. A 52-item patient questionnaire (the “Paroxysmal Event Symptoms Questionnaire” [PESQ]) comprises 3 demographic questions (age, sex, years of formal education), 14 questions regarding medical history, and 35 questions of peri-ictal symptoms. An 18-item witness questionnaire (“Paroxysmal Event Witness Questionnaire” [PEWQ]) comprises 18 questions regarding ictal semiology. Participants/witnesses could complete the PESQ/PEWQ either online (through a dedicated interface, hosted on a secure university server) or on paper.

We provide PESQ and PEWQ in eAppendix 1.

### Diagnostic Reference Standard

Because we sought to recruit an unselected first-presentation TLOC cohort, we were unable to use gold standard diagnoses as reference; most of the people with epilepsy or FDSs do not have sufficiently frequent seizures to capture ictal EEG recordings^6^ while ESC guidance supports making clinical diagnosis without further investigation of uncomplicated vasovagal syncope.^27^ However, consensus clinical diagnosis of TLOC-causing disorders by multiple experts is highly reliable.^28,29^ We, therefore, use as reference standard the consensus diagnosis reached by 2 independent TLOC experts (M.R. and S.J.H.), blinded to PESQ and PEWQ data (questionnaire responses and classifier predictions), from notes review at least 6 months after enrollment.

Participants for whom no firm clinical diagnosis could be reached at the end of follow-up, or who had multiple diagnoses, were excluded from further analysis. A previous study of patients with suspected seizures in this population found that only 1.1% of patients could not be given a single etiologic diagnosis, so we anticipated a low diagnostic failure rate.^24^

### Analysis

#### Development

We used an iterative feature selection algorithm to identify most highly discriminatory features from the PESQ data provided by the first 100 participants. The details of this stage of the analysis are the same as that used in our previous initial development research.^18^ An RF trained using the CART algorithm on all development data (5,000 iterations, sampling √*p* predictor variables [where *p* = number of predictors] at each iteration) ranked the relative prediction importance of each predictor variable; progressively smaller RFs are then trained by removing the least important 20% of predictors and calculating out-of-bag prediction error ([OOBE]; average of the prediction error of each tree in the ensemble for data not sampled in its training, an estimate of generalization error equivalent to cross-validation methods^30^) of the resulting RF. The final set of predictor variables and RF used is that which minimizes OOBE; we previously found that more parsimonious feature selection resulted in significant impairment in performance.^31^ The final prediction model was an RF trained on all development data using the selected set of predictor variables. We selected hyperparameters on the basis of optimization in our pilot study, combined with evidence that classifier performance is robust to variations in these hyperparameter settings.^32^

To compare a nonlinear machine learning–based classifier against more traditional regression approaches, we also trained a penalized maximum likelihood (LASSO) classifier, choosing the model complexity/regularization parameter λ that minimized the cross-validated mean-squared error.

#### Validation

We validated prediction models against an independent validation data set. Both RF and regression models classified participants into likely diagnoses of epilepsy, syncope, or FDSs, evaluating performance regarding overall classification accuracy, as well as sensitivity, specificity, positive predictive value (PPV), and negative predictive value (NPV).

Given that syncope due to structural or arrhythmic cause is the condition with highest short-term morbidity/mortality, we determined sensitivity for syncope to be our primary outcome. We compared sensitivity for syncope of the new RF with that found in our initial research^18^ (χ^2^ test, one-tailed α = 0.05 for target sensitivity 97.5%). We also tested the hypothesis that classification accuracy of the RF is significantly greater than that of the regression model (McNemar test, α = 0.1).

Furthermore, we performed a post hoc analysis simulating performance of the classifier as a clinical decision aid (CDA) augmenting the initial assessing clinician's evaluation. For this, classifier diagnosis was used as “tie-break” for occasions when the initial assessing clinician gave no working diagnosis, otherwise the initial diagnosis was used; these diagnoses were compared with our reference standard.

### Standard Protocol Approvals, Registrations, and Participant Consents

We pre-registered the study protocol on clinicaltrials.gov (ID: NCT05367999). Ethical approval was obtained from NHS Health Research Authority Edgbaston Research Ethics Committee (IRAS: 304114). All patients and witnesses confirmed their consent to participate before completing the PESQ/PEWQ and were able to withdraw at any time.

### Data Availability

Deidentified data set, data dictionary, and analytic codes are available on request from the authors.

## Results

### Descriptive Results

#### Screening and Recruitment

**Figure 1:**
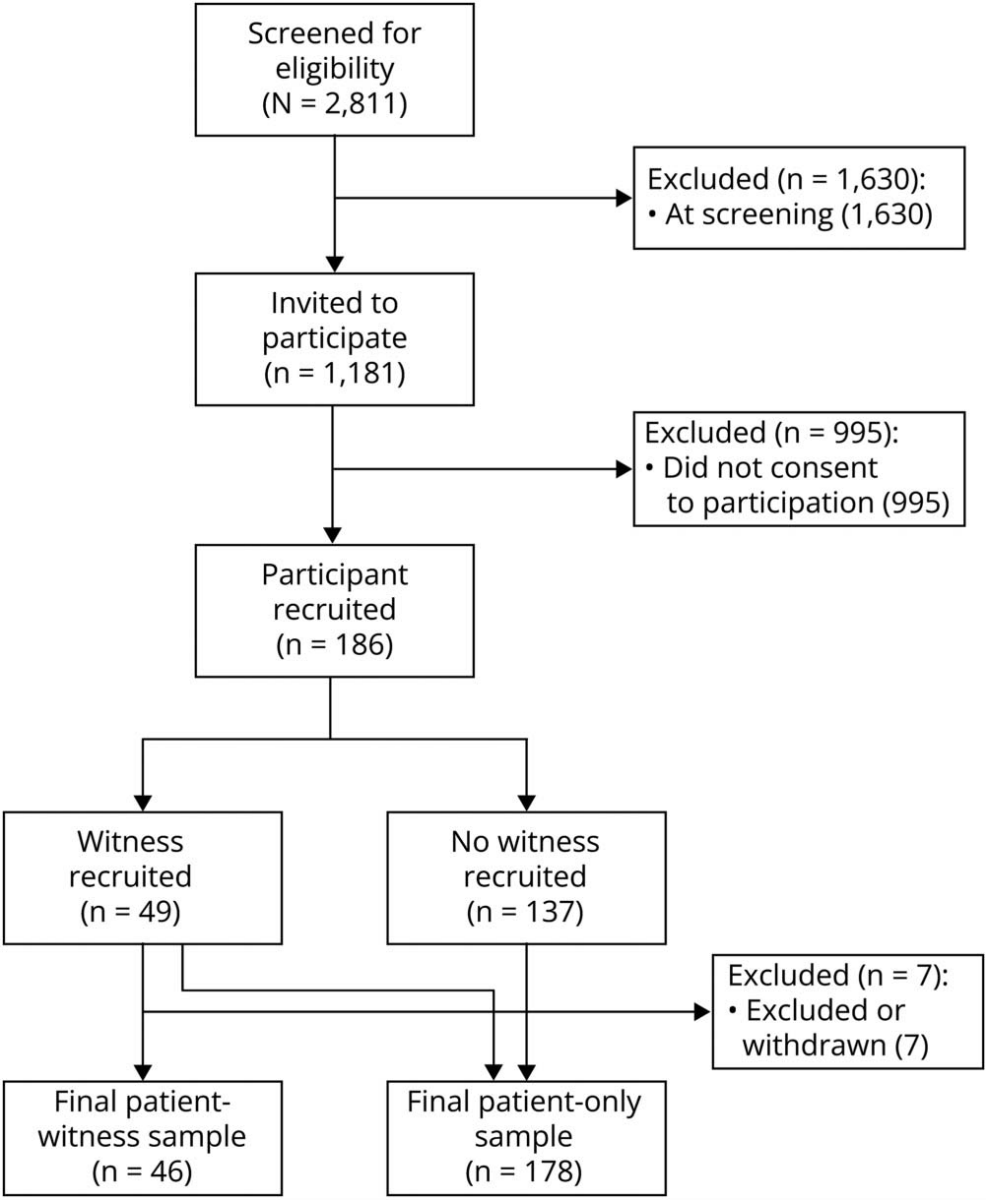
Participant Flow Diagram

Of 2,811 patients screened for recruitment, 1,181 were approached to participate. Of these, 186 (15.7%) gave consent and completed the PESQ. Seven participants either withdrew or were deemed ineligible at the end of follow-up. One participant was excluded because no final diagnosis could be reached. Therefore, we included 178 participants in further analyses; the first 100 participants constituted the development data set and the remaining 78 validation. Of 178 included participants, 46 identified a witness who completed the PEWQ. Figure 1 illustrates participant flow through the study. Patients were deemed ineligible at screening most commonly because of not experiencing TLOC (592%, 36.3%), having previous specialist care for a TLOC-causing disorder (352%, 21.6%), or having a secondary cause for their TLOC (285%, 17.5%). Only 59 (3.6%) were ineligible for inability to complete the PESQ because of language or other barriers.

#### Demographics and Diagnoses

Syncope was the most common final diagnosis (134 participants; 75.3%), followed by epileptic seizure (32 participants, 18.0%). Table 1 summarizes participant demographics.

**Table 1:** Summary of Participant Demographics

Diagnosis	N (% total)	Median age (range)	N (%) female	N (%) providing witness
Syncope	134 (75.3)	64 (17–94)	75 (56.0)	34 (25.4)
ES	32 (18.0)	47.5 (16–86)	14 (43.8)	11 (34.4)
FDS	12 (6.7)	31 (16–57)	9 (75.0)	1 (8.3)

Abbreviations: ES = epileptic seizure; FDS = functional/dissociative seizure.

Expert raters agreed on diagnoses in 144 of 178 cases (80.1%). For the remainder, consensus diagnoses were reached by discussion. One participant was excluded because of persistent uncertainty regarding diagnosis.

Expert raters agreed with the initial clinician diagnosis in 120 of 178 cases (67.4%).

#### Patient Questionnaire

Most frequently endorsed PESQ items across all participants were “I want to know what has happened when I black out” (140 participants), a history of light-headed spells (88 participants), and “I feel hot or cold in my attacks” (80 participants). Least frequently endorsed items were “The sight of blood or needles triggers my attacks,” “During my attacks I have memories of a past bad experience which I cannot stop,” and a history of brain tumor (each 4 participants).

**Figure 2:**
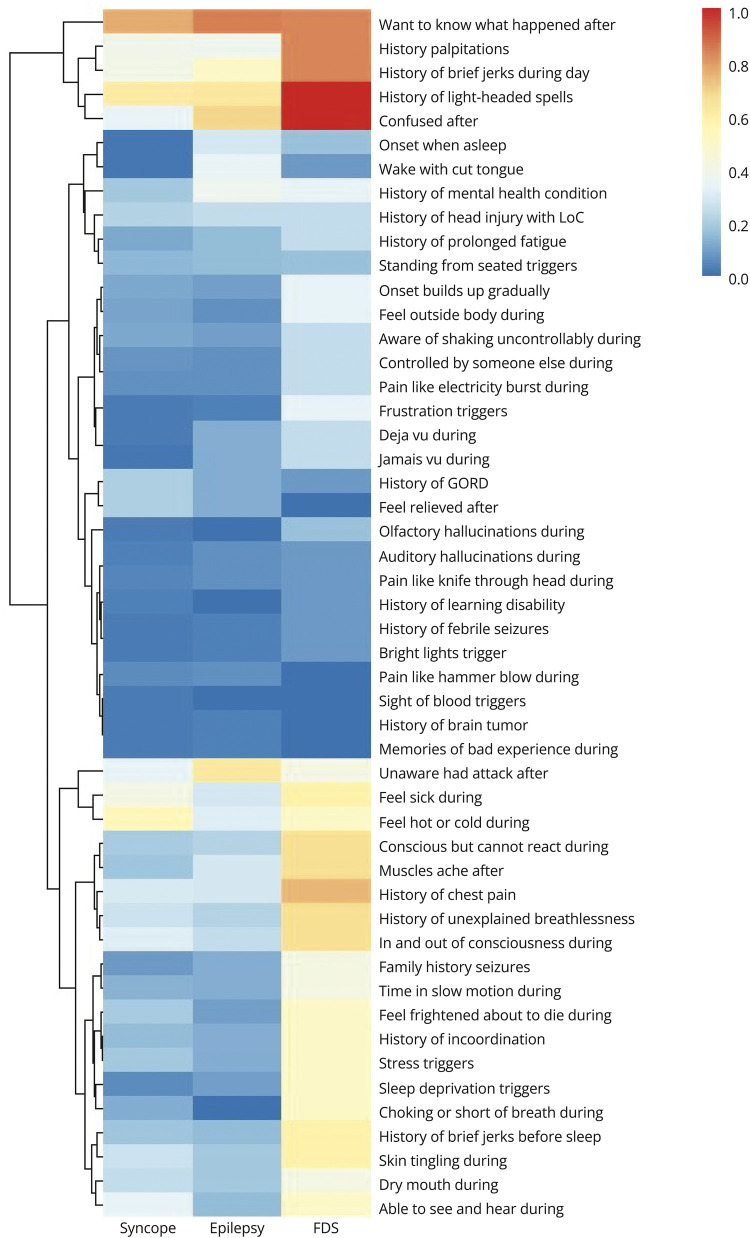
Heatmap of Relative Frequency of Patient-Reported Symptoms by Diagnosis Color depicts the proportion of respondents with each diagnosis endorsing each item. FDS = functional/dissociative seizure. Dendrogram on the left-hand side demonstrates hierarchical clustering of questionnaire items (items that are most likely to co-occur are clustered; then, clusters of items most likely to co-occur clustered on higher levels).

Figure 2 presents relative proportions of participants endorsing each PESQ item by final diagnosis, with hierarchical clustering of questionnaire items.

#### Witness Questionnaire

A total of 46 witnesses completed the PEWQ. Of these, only one was for a participant with FDSs. Most frequently endorsed items were “The skin or lips looked pale during the attack” (33 witnesses), “During the attack, arms and legs are limp” (31 witnesses), and “Breathing was shallow or quiet after the attack” (28 witnesses). Least frequently endorsed were “The attacks involve violent thrusting of the hips” (2 witnesses), “The attacks involve chewing, smacking, or licking movements of the mouth and lips” (4 witnesses), and “The attacks involve scratching or bicycling movements of the legs” (4 witnesses).

**Figure 3:**
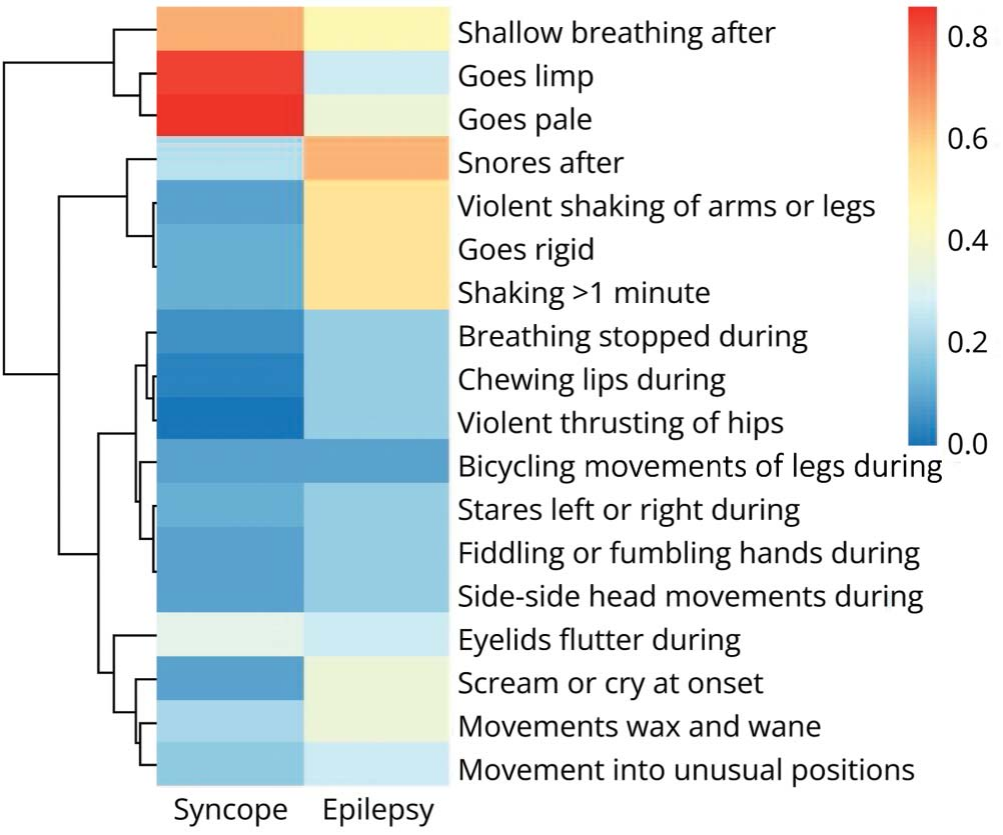
Relative Frequency by Diagnosis of Witness Reports Color depicts the proportion of witnesses for respondents with each diagnosis endorsing each item. Dendrogram to the left depicts hierarchical clustering of questionnaire items (items that are most likely to co-occur are clustered; then, clusters of items most likely to co-occur clustered on higher levels). FDS not shown because only a single PEWQ was completed for participants with this diagnosis. FDS = functional/dissociative seizure; PEWQ = Paroxysmal Event Witness Questionnaire.

Figure 3 presents relative proportions of witnesses endorsing each PEWQ item by diagnosis; because only 1 participant with FDSs identified a witness, results are shown for syncope and epileptic seizures only.

Hierarchical clustering of PEWQ items (displayed in the dendrogram in Figure 3) identifies 2 high-level clusters of “syncopal” features (shallow breathing, flaccidity, pallor) and “other” features. The “other” features subcluster into a group highly reported in epileptic seizures (postictal stertor, violent shaking, rigidity, and prolonged shaking) and a group of less frequently reported symptoms.

### Sensitivity and Specificity of Individual Questionnaire Items

No item was more than 80% sensitive and specific for any diagnosis. Three PESQ items had individual sensitivity and specificity >0.5 for either syncope or epileptic seizures. More met this criterion for FDS, but given low FDS prevalence, PPV/NPV did not exceed 0.2.

Seven PEWQ items had individual sensitivity and specificity >0.5 for either syncope or epileptic seizures.

The most sensitive individual question for syncope with adequate specificity was “The skin or lips looked pale during the attack” (sensitivity = 85.3%), and the most specific with adequate sensitivity was “During the attack, arms and legs are limp” (specificity = 72.7%). For epilepsy, the most sensitive item from the PESQ was “After my attacks I feel very confused” (68.8%), while the most specific was “The attacks involve violent shaking of the arms or legs” (91.2%) from the PEWQ.

Table 2 summarizes diagnostic performance of most highly discriminating individual items from the PESQ and PEWQ; eTable 1 provides complete data for all items.

**Table 2:** Predictive Performance of Most Highly Discriminating Individual PESQ and PEWQ Items

Final diagnosis	Item	Sensitivity	Specificity	PPV	NPV
Syncope					
Patient	Hot or cold during	0.537	0.636	0.818	0.689
Witness	Limp during	0.824	0.727	0.903	0.571
	Pale during	0.853	0.636	0.878	0.583
	Shallow breathing after	0.647	0.545	0.814	0.333
Epilepsy					
Patient	Confused after	0.688	0.603	0.275	0.102
	Unaware had attack	0.625	0.658	0.286	0.111
Witness	Violent shaking of arms or legs	0.545	0.912	0.667	0.861
	Arms and legs rigid	0.545	0.882	0.600	0.857
	Shaking >1 min	0.545	0.882	0.600	0.857
	Snoring after	0.636	0.736	0.438	0.862

Abbreviations: NPV = negative predictive value; PESQ = Paroxysmal Event Symptoms Questionnaire; PEWQ = Paroxysmal Event Witness Questionnaire; PPV = positive predictive value.

### Model Development

#### PESQ Only

Because PESQ and PEWQ were available for only 46 participants, we performed model development using PESQ responses only. eFigure 1 demonstrates relative predictor importance of PESQ responses. Some items contributed heavily to classifier performance (e.g., “My attacks come on when I am asleep” and “I wake from my attacks with a cut tongue”) while others decreased performance (e.g., “I want to know what has happened when I black out” and “history of light-headed spells”). Historical variables showed generally lower predictor importance than peri-ictal symptoms.

**Figure 4:**
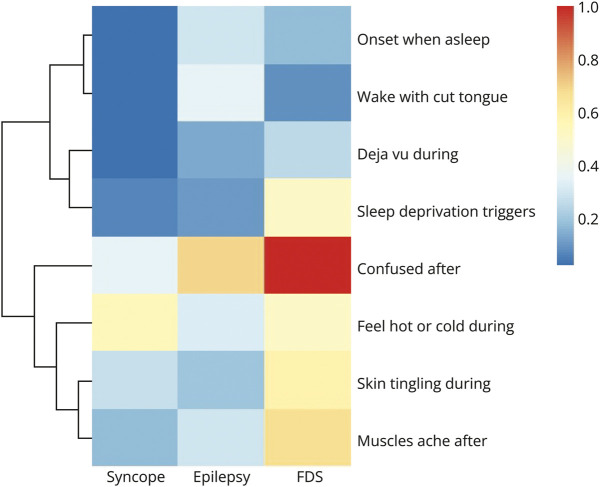
Relative Reporting Proportions of Predictors Included in PESQ Classifier FDS = functional/dissociative seizure; PESQ = Paroxysmal Event Symptoms Questionnaire.

Feature selection identified a 9-variable RF as optimal: it used 1 demographic variable (age in years) and 8 symptoms. Figure 4 shows these symptoms and relative reporting proportions by diagnosis. Hierarchical clustering demonstrates 2 high-level clusters within these items, a “seizure” cluster (onset from sleep, waking with cut tongue, deja vu, sleep deprivation trigger) and a “polysymptomatic” cluster, highly reported in FDSs.

The optimal RF had an OOBE of 0.21 (79% accuracy).

Penalized regression (LASSO) identified 7 predictors with nonzero coefficients: 1 demographic (age in years), 1 historical (brief jerks during the day), and 5 symptoms (“My attacks come on when I am asleep”; “I wake from my attacks with a cut tongue”; “I feel hot or cold in my attacks”; “After my attacks I feel very confused”; and “During my attacks I smell things that are not really there”). eTable 2 provides model coefficients, and eFigure 2 presents the hyperparameter tuning.

#### PESQ-PEWQ

Given the small PESQ-PEWQ data set, we used all available data for model development. Because only 1 participant in this data set had FDSs, they were excluded from the analysis and a binary classifier for distinguishing syncope from epileptic seizures was developed. eFigure 3 demonstrates predictor importance for the combined PESQ-PEWQ classifier. Again, symptom and witness reports were the most important predictors, with the latter disproportionately represented among the most important predictors.

The optimal model used 3 predictor variables: 2 symptom reports from the PESQ (“My attacks come on when I am asleep” and “I wake from my attacks with a cut tongue”) and one from the PEWQ (“During the attack, arms and legs are limp”).

### Model Validation

#### PESQ-Only

To avoid overfitting, we performed external validation using a separate holdout data set comprising the last 78 recruited participants. The PESQ classifier correctly identified 63 of 78 diagnoses (80.8%; 95% CI 70.0–88.5). The sensitivity for syncope was 96.6% (87.0–99.4). Table 3 summarizes the diagnostic performance for each diagnosis. eTable 3 gives the confusion matrix.

**Table 3:** Diagnostic Test Statistics for the PESQ Classifier

	Sensitivity	Specificity	PPV	NPV
Syncope	0.966 (0.870–0.994)	0.400 (0.200–0.636)	0.824 (0.708–0.902)	0.800 (0.442–0.965)
Epilepsy	0.429 (0.188–0.704)	0.969 (0.882–0.995)	0.750 (0.356–0.956)	0.886 (0.782–0.946)
FDS	0.167 (0.009–0.635)	0.986 (0.915–0.999)	0.500 (0.095–0.905)	0.934 (0.847–0.976)

Abbreviations: FDS = functional/dissociative seizure; PESQ = Paroxysmal Event Symptoms Questionnaire; PPV = positive predictive value.

Values in brackets represent 95% CIs. Syncope sensitivity denotes the proportion of patients diagnosed with syncope after 6-mo follow-up who were classed as syncope based on the initial questionnaire at first presentation and the PESQ classifier. Syncope specificity denotes, among those participants who (after 6 mo) were given a nonsyncope diagnosis, the proportion of those who were also given a nonsyncope diagnosis using the PESQ classifier at first presentation. Syncope PPV denotes the proportion of those classified as syncope who received a syncope diagnosis at follow-up. Syncope NPV denotes the proportion of those classified as nonsyncope who received a nonsyncope diagnosis at follow-up. The same applies mutatis mutandis for epilepsy and FDS.

The classifier did not differ significantly from our prespecified target sensitivity for syncope of 97.5% (*p* = 0.644). We also performed a post hoc analysis comparing classifier diagnosis with the initial assessing clinician's diagnosis and referrer or discharge diagnosis. The classifier accuracy was numerically, but not statistically significantly, superior to initial diagnosis (accuracy 70.5%; *p* = 0.192) and referrer diagnosis (accuracy 75.6%; *p* = 0.561).

We hypothesized that machine-learning classifiers such as RFs would outperform regression modeling because they allow for items to have different contributions to differential diagnosis depending on co-occurrence with other items. We, therefore, compared performance of the PESQ classifier with a linear model (penalized regression model). The latter classified 58 of 78 diagnoses (74.4% [63.0–83.3]) correctly. The performance was, therefore, worse than the PESQ classifier, but this difference was not statistically significant (*p* = 0.499). We provide the confusion matrix (eTable 4) and diagnostic statistics (eTable 5) for this model in the supplementary materials.

To assess whether the RF classifier development described in our prespecified protocol could be improved on using other machine-learning methods and models, we describe elsewhere a post hoc analysis using ensemble comparison of multiple machine-learning approaches using H_2_O AutoML. However, this did not produce a model that outperformed our RF classifier.^33^

#### PESQ-PEWQ

There was no separate validation data set for the PESQ-PEWQ classifier. Evaluating model performance using training data will overestimate model performance because of overfitting; we, therefore, use OOBE (equivalent to cross-validation methods).^30^

OOBE was 11.1%, equivalent to a classification accuracy of 88.9%. eTable 6 provides the confusion matrix for this classifier.

#### Pilot iPEP Classifiers

The patient-only and patient-witness iPEP classifiers from our pilot study performed worse than the new models in this external validation data set, classifying 75.8% (68.8–81.8) and 78.3% (63.2–88.5) of diagnoses correctly. We provide full details in eAppendix 2, given the confusion matrix and diagnostic test statistics for the patient-only (eTables 7 and 8, respectively) and patient-witness (eTables 9 and 10) iPEP classifiers.

### PESQ-Augmented Clinician Performance

Using PESQ classification to adjudicate in cases where the initial assessing clinician gave no diagnosis, or one unlikely to cause TLOC (e.g., TIA), then classifier-augmented clinical decision agreed with experts on 66 of 78 patients (accuracy = 84.6% [95% CI 74.3–91.5]).

When comparing classifier performance with that of the initial clinician, the initial clinician made the correct diagnosis in 8 cases in which the classifier was incorrect; 6 of these were epileptic seizures and 2 syncope. In 16 cases in which the initial clinician misdiagnosed the patient or made no diagnosis, the classifier was correct; 15 of these were syncopal and 1 FDS.

## Discussion

This study provides evidence that a machine learning–based classifier solely using patients' own responses to a brief patient-completed questionnaire can identify common causes of TLOC with an accuracy greater than 80%. In this study, only 67.4% were correctly diagnosed in emergency or primary care at the point of presentation or received no probable diagnosis on initial assessment and 76.4% after further nonspecialist assessment. While overall performance is insufficient to recommend its routine clinical use, comparison with the present standard of care does suggest that such a tool may helpfully augment present unstructured clinical assessment and an illustrative post hoc analysis suggests that classifier augmentation could improve the diagnostic accuracy of initial clinical diagnoses to 84.6%.

There is increasing recognition that artificial intelligence and machine learning can be used to augment (rather than replace) clinicians' decisions in this fashion.^34^ With estimated annual (direct and indirect) costs of epilepsy misdiagnosis in England and Wales running to £138 million,^35^ this represents potential for significant cost savings and patient benefit.

The PESQ classifier is unusual in providing three-way classification including all the common primary causes of TLOC. Candidate clinical decision rules for discriminating between syncope and bilateral tonic-clonic seizures,^15,36^ or epilepsy and FDSs,^16,26,37^ focus on the mathematically simpler problem of binary classification. Our classification problem more accurately reflects the challenge faced by the primary/emergency care clinician.

Our results underscore the importance of holistically evaluating clusters of clinical features in the differential diagnosis of TLOC, rather than treating individual features in isolation as pathognomonic. No single PESQ or PEWQ item proved both sensitive and specific for any diagnosis; furthermore, a classifier that evaluated combinations of features nonlinearly performed better than a linear model in identifying the correct diagnosis (although this did not reach statistical significance).

Our results also underscore the utility of systematic interrogation of ictal experience. Peri-ictal symptoms were more discriminating than participants' medical histories, highlighting the importance of thoroughly exploring TLOC experiences. Previous work suggests that combining open questions with systematic, prompted questioning about ictal experience (e.g., through questionnaires such as the PESQ) identifies more ictal symptoms than open questions alone^38,39^; the effect of prompting may differ between diagnoses.^26,40^

A major limitation of this study is the low witness recruitment. Just 46 of 178 participants (25.8%) identified a witness able to complete the PEWQ. We could, therefore, not both develop and independently validate a classifier based on both PESQ and PEWQ. This reflects a common difficulty: while professional guidance stresses the importance of obtaining a witness report for patients with TLOC,^41^ clinicians may struggle to achieve this in practice. Further work should identify means of supporting witness identification and questioning to aid TLOC differential diagnosis. In our previous study involving patients with long-standing TLOC disorders, witness information was available from 83% of participants^17^; initial presentations may be less likely to be observed (or observed by those close to the patient). Given the low number of witnesses recruited and our consequent inability to provide independent validation of the combined PESQ-PEWQ classifier, the results for this classifier should be considered exploratory only.

Only 15.7% of eligible presentations were recruited to our study. If there were systematic differences in recruitment by diagnosis (or e.g., patient demographics), this would bias the external validity of our results. Further work should address barriers to recruitment in such studies (which in principle place minimal burden on participants, and evidence from our qualitative work suggests that the interventions were highly acceptable to participants).

Our reference standard diagnosis does not represent the gold standard for any of our target conditions. This places an obvious caveat to our estimations of classifier performance because the reference is a “best possible” diagnosis rather than a clinically definite one. This is underlined by the fact that our expert raters either initially disagreed on or needed discussion to arrive at a consensus diagnosis for 34 of 178 participants (19.1%). However, this was a necessary compromise to ensure the ecological validity of our sample; most of the people presenting with TLOC will not achieve a gold standard diagnosis—for example, their attacks will be too infrequent (or even one-off) to be witnessed by an expert or captured on video-EEG or cardiac monitoring. We intend to follow up clinical outcomes for our participants to evaluate the stability of “best possible” diagnoses over the longer term and the impact this may have on validity of our results.

We did not achieve our target validation sample size; because prevalence of syncope was higher than expected, the study was nonetheless adequately powered for our primary outcome. However, the sample was smaller than that recommended in simulation studies that estimate empirical sample size requirements for external validation of multivariable prognostic models.^42^ The high prevalence of syncope in our sample is striking, differing from estimates reported elsewhere.^23,43^ This may reflect the true incidence of the respective diagnoses^2,12,44^; however, it may be that people with epilepsy and FDS—still stigmatized conditions^45,46^—were less willing to participate. It is a strength of this study that not only recruitment included patients referred to specialist clinics, but that we were also able to recruit 39 participants diagnosed with reflex syncope (the single most common cause of TLOC^23^) who were discharged directly from ED or AMU with no further assessment. This population would not have been captured in specialist clinics.

We demonstrate that a simple, patient-completed questionnaire can provide relevant information to differential diagnosis of TLOC but cannot replace clinician assessments. Rather than automated assessment, such tools may best have a role in augmenting clinician evaluation, as CDAs.^34^ This would allow the clinician to combine outputs with their own holistic assessment to determine a working diagnosis.

Patient-completed tools to reduce clinician workload are not frequently used, despite demonstrated feasibility of patient application of CDAs for their own care.^47,48^ Given that around 9 in 10 patients attending EDs in England in 2018–2019 spent over an hour in the department,^49^ ED attendances provide ample opportunity for self-administration. Implementation within now-commonplace smartphones or browser-based applications could ensure user friendliness. Combining machine-learning predictions with tools such as locally intelligible model-agnostic explanations^50^ to render them more explainable to patients and clinicians may help clinicians to feel their use more defensible, overcoming objections to their otherwise “black-box” nature.^33^ Together, these considerations should address the main factors affecting ED clinicians' willingness to use CDAs in their clinical practice.^51^ Patient-completed tools need not be restricted to simple questionnaires as we have used; in other works, we have also demonstrated feasibility of automated capture and analysis of patients' spoken accounts^52^; these 2 approaches could be combined. Further research on clinician experience of interacting with such a CDA could support wider use.

Misdiagnosis leads to ineffective, potentially dangerous, treatment.^8,10^ Diagnoses such as epilepsy also have psychological and social implications, such as for employment and driving.^35^ Methods to support accurate, prompt diagnosis with efficient use of medical resources—both human (e.g., referral to the appropriate specialist team) and investigative (reducing requesting of inappropriate low-yield investigations such as chest or brain imaging for syncope)^12^—are needed to ensure efficient and effective management of this common and disruptive presentation. There is also a need for work clarifying the human and economic costs of missed or delayed diagnoses to support economic modeling of benefits of implementation of CDAs.

## Glossary

AMU: acute medical unit
CDA: clinical decision aid
ED: emergency department
ESC: European Society of Cardiology
FDS: functional/dissociative seizure
iPEP: initial PEP
NPV: negative predictive value
OOBE: out-of-bag prediction error
PEO: Paroxysmal Event Observer
PEP: Paroxysmal Event Profile
PESQ: Paroxysmal Event Symptoms Questionnaire
PEWQ: Paroxysmal Event Witness Questionnaire
PPV: positive predictive value
RF: random forest
TLOC: transient loss of consciousness
